# Hydrogen Production from Ammonia Electrochemical Cracking

**DOI:** 10.1002/advs.78256

**Published:** 2026-10-11

**Authors:** Yike Ye, Yi Shun Lim, Qian Wu, Kamal Elouarzaki, Zhichuan J. Xu

**Affiliations:** ^1^ Nanyang Environment and Water Research Institute (NEWRI) Interdisciplinary Graduate Programme 1 Cleantech Loop Nanyang Technological University Singapore Singapore; ^2^ School of Materials Science and Engineering Nanyang Technological University Singapore Singapore; ^3^ Center for Advanced Catalysis Science and Technology Nanyang Technological University Singapore Singapore

**Keywords:** ammonia oxidation reaction, electrocatalysis, H_2_ carrier, NH_3_ cracking, techno‐economic analysis

## Abstract

Utilizing NH_3_ as a H_2_ carrier has been proposed to address the storage and transportation bottlenecks of the green hydrogen supply chain. NH_3_ thermal cracking is a conventional technique for H_2_ recovery after transport, but its infrastructure‐intensive nature limits its application in decentralized H_2_ production scenarios. Consequently, ammonia electrolysis, coined “e‐cracking” in this article, is proposed as a complementary technique for such scenarios. This emerging technique couples hydrogen evolution reaction (HER) with ammonia oxidation reaction (AOR), which was previously studied in the context of ammonia fuel cells. While thermal cracking has been extensively investigated regarding catalyst development and economic feasibility, e‐cracking is at an early stage and lacks such efforts, as reflected by its lower technology readiness level (TRL). To bridge this gap, this Perspective provides a comprehensive analysis of e‐cracking addressing both aspects. We begin with an overview of catalyst development for both techniques and then conduct a techno‐economic analysis (TEA) based on reported performance data. By comparing the levelized cost of hydrogen (LCOH), we demonstrate e‐cracking's theoretical potential for lower costs and identify the current limitations arising from catalytic performance. Finally, we provide an outlook on research directions and strategies to advance NH_3_ e‐cracking toward practical implementation.

## Graphical abstract

NH_3_ electrochemical cracking (e‐cracking) is a complementary technique to centralized thermal cracking for releasing H_2_ from NH_3_. In this article, we review recent advances in catalyst development for these cracking techniques and conduct a techno‐economic analysis (TEA) to evaluate the economic feasibility of e‐cracking. Finally, we outline key challenges and opportunities for the development of NH_3_‐based hydrogen infrastructure.

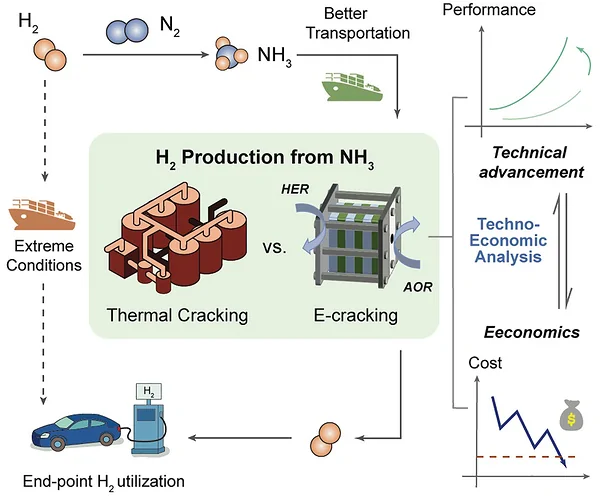

## 1 Introduction

The rising concerns of climate changes caused by increasing fossil fuel consumption prompt the transformation to renewable energy sources, such as solar and hydro power [1]. However, these energy sources may fail to provide a stable energy supply due to their intermittency and variability [2]. Therefore, after harvesting energy from these renewable sources, it is necessary to convert the energy into carriers for storage, transportation, and further utilization. Among energy carriers, H_2_ has attracted much attention due to its carbon‐free nature and high mass energy density [3]. The extensive utilization of hydrogen is essential for reducing the dependence on fossil fuels and thereby achieving decarbonization. Nevertheless, establishing global H_2_ supply chains remains challenging because of the extreme conditions of H_2_ storage and transportation. Since the density of H_2_ is low, little H_2_ can be stored at ambient conditions. Consequently, during these processes, H_2_ must be compressed (200–700 bar at room temperature) or liquefied (‐253°C at 1 atm), resulting in high cost, low efficiency, and safety concerns [4]. It is estimated that the liquefaction from the condition of 20 bar and 300 K consumes over 11 kWh kg_H2_^−1^, which approaches 1/3 of H_2_ low heat value (LHV) [5]. Moreover, hydrogen embrittlement and the risk of explosion raise safety issues and hinder the development of H_2_ supply chains [6]. To address these problems, H_2_ is converted into chemicals, termed H_2_ carriers, for more convenient and economical transportation. After arriving at destinations, these chemicals are decomposed to release H_2_ for further distribution and utilization.

NH_3_ is one of the most promising H_2_ carriers due to its high volumetric H_2_ density, milder operating conditions, and carbon‐free nature [7]. The volumetric H_2_ density of NH_3_ reaches 120.3 kg_H2_ m^−3^, nearly twice that of liquid H_2_. Additionally, NH_3_ can be liquefied under much less stringent conditions than H_2_, requiring only 1 bar at −33°C or 10 bar at 20°C [8]. Consequently, using NH_3_ as an H_2_ carrier markedly reduces energy cost and minimizes safety risks. Moreover, the economic feasibility of this H_2_ carrier strategy has been quantitatively assessed by techno‐economic analysis (TEA). For example, the International Energy Agency (IEA) evaluated the costs of transmission and storage of hydrogen gas and NH_3_. At a pipeline transmission distance of 1,000 km, using NH_3_ costs only $0.25 kg_H2_^−1^, half the cost for using H_2_. Furthermore, when transporting 2,500 km, the cost difference between transporting NH_3_ and H_2_ exceeds $2 kg_H2_^−1^, the cost of conversion from H_2_ to NH_3_, which highlights the advantages of NH_3_ for long‐term transmission. In terms of maritime transport, H_2_ must be liquefied. As a consequence, the capital expense (CAPEX) for the required ships is almost 5 times higher than that for NH_3_ ships [9, 10]. The remarkable decrease in transportation costs underlines the significance of employing NH_3_ as an H_2_ carrier.

Additionally, NH_3_, as fertilizers and industrial chemicals, has already been traded, transported, and distributed worldwide. The Haber‐Bosch process, a method for industrial‐scale NH_3_ synthesis, has been well developed. Besides, current worldwide infrastructures for NH_3_ transmission and storage have been developed. For instance, the United States has a NH_3_ pipeline network longer than 3,000 km [11]. By leveraging these facilities and related technologies, employing NH_3_ as an H_2_ carrier on a large scale becomes feasible. Moreover, with the rising consensus of decreasing carbon footprint, more and more countries have launched and/or announced projects for international trades of low‐emission NH_3_ (Figure 1a). Saudi Arabia has shipped over 0.1 million tons NH_3_ to China, the Europe Union, etc. in 2023, and all these trades have been verified as low‐carbon by TÜV Rheinland [12]. Notably, the traded NH_3_ is no longer solely used as fertilizers, but also gradually gaining attention as an energy carrier (green fuel) or H_2_ carrier. Chile, known for its abundant renewable energy sources, is planning to establish a 600,000‐ton‐per‐year renewable NH_3_ plant in Mejillones [13]. Envision Energy's renewable ammonia plant in Inner Mongolia, China, which produces 320,000 tons per year, completed its first certified RFNBO‐compliant shipment to South Korea in early 2026 [14]. On the import side, Japan, driven by ambitious hydrogen strategies, aims to import low‐carbon NH_3_ from Australia, the United States, etc. for energy applications. These developments underscore the pivotal role of NH_3_ as an H_2_ carrier in the global clean energy landscape. However, to fully unlock its potential remains challenging, as NH_3_ cracking for H_2_ recovery is still insufficiently explored.

**FIGURE 1:**
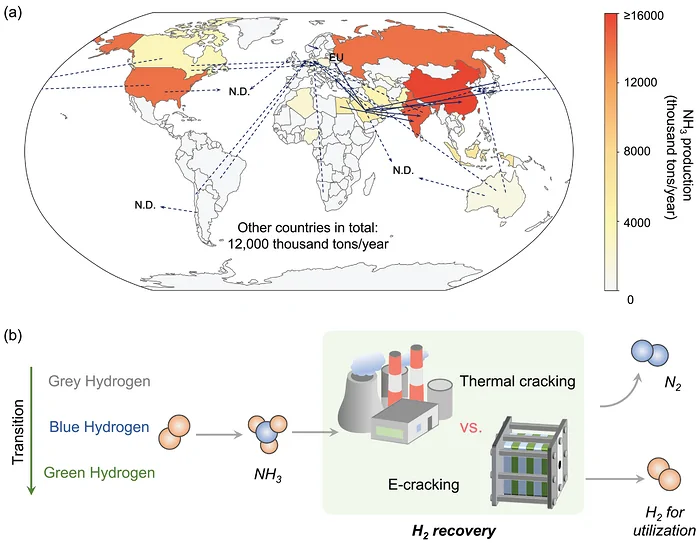
(a) World NH_3_ production (in thousand metric ton) in 2025 by country with current (solid line) and announced (dashed line) projects of international low‐emission NH_3_ trades. Data were obtained from Ref.  [12, 20]. N.D.: Destination not defined. (b) Illustration of the role of NH_3_ in hydrogen economy.

During NH_3_ cracking, NH_3_ is decomposed, and H_2_ is released for subsequent utilization (Figure 1b). Till now, in industry, this is predominantly conducted through thermal catalysis, where NH_3_ breaks down into N_2_ and H_2_ following the reverse reaction of Haber‐Bosch process. The process is also termed thermal cracking. To ensure a high conversion of NH_3_, the reaction usually proceeds at high temperatures [15, 16]. Contingent on catalysts, the reaction temperature varies from 500°C to 900°C [17]. Noble metal‐based catalysts (e.g., Ru‐based) generally operate at lower temperature, while the cheaper transition metal‐based catalysts (e.g., Ni‐based and Fe‐based catalysts) require higher temperature. In addition to the thermal catalytic process, NH_3_ cracking can be achieved through electrochemical procedures, which we coin electrochemical cracking (e‐cracking), similar to methanol electrochemical reforming (e‐reforming) [18]. In e‐cracking, aqueous NH_3_ undergoes electrolysis involving two half‐reactions: ammonia oxidation reaction (AOR) and hydrogen evolution reaction (HER). H_2_ is produced and collected at the cathode for further utilization. Since e‐cracking operates under ambient conditions, it is suitable for flexible and decentralized applications. However, the lack of robust AOR catalysts poses a challenge to the implementation of e‐cracking. Specifically, unlike the well‐established HER catalysts, highly efficient and stable AOR performance has yet to be achieved, primarily due to severe surface poisoning. This bottleneck persists despite decades of historical research driven by direct NH_3_ fuel cells, which were proposed as an alternative to H_2_ fuel cells in the late 1960s for improved fuel storage and distribution conditions [19]. Nevertheless, the novel combination of AOR and HER offers new opportunities for realizing flexible and green H_2_ production, calling for renewed efforts in AOR catalyst development.

Beyond catalyst development, a rigorous assessment of economic feasibility is required for practical implementation. While this has been investigated for the relatively mature NH_3_ thermal cracking [21, 22], such efforts toward e‐cracking remain scarce. This gap stems from the relatively recent emergence of the e‐cracking technique, coupled with the limited understanding of its catalytic and operational challenges. In this Perspective, we compare these two NH_3_ cracking routes and provide insights into the future implementation of these techniques in H_2_ economy. We first elucidate the extensive research on catalyst development for thermal cracking and AOR. We then conduct TEA to assess the market viability of these cracking approaches. By comparing levelized cost of hydrogen (LCOH) for both routes, we reveal the current insufficiency of AOR catalyst development and the significant potential for reducing LCOH through mitigating surface poisoning in e‐cracking. Finally, we provide perspectives on catalyst development, device design, greener NH_3_ production, and international political strategies, which could advance the implementation of NH_3_ e‐cracking and accelerate the transition to H_2_ economy.

## 2 NH_3_ Cracking Process

### 2.1 Thermal Cracking

Currently, the major industrial applications of NH_3_ thermal cracking are annealing metals and galvanizing. NH_3_ is decomposed into H_2_ and N_2_, which provide a reductive environment and prevent metal oxidation [23]. Initially, thermal cracking was catalyzed by the same catalysts used in the Haber‐Bosch process, i.e., Fe‐based catalysts and Ru‐based catalysts. Thereafter, Ni‐based catalysts emerged as attractive candidates with outstanding performance and low costs. Till now, Ni catalysts supported on Al_2_O_3_ (Ni/Al_2_O_3_) have been applied commercially for thermal cracking [24]. In the past years, with the development of H_2_ economy, thermal cracking has been gradually employed to produce very high purity H_2_ for energy generation, such as in proton exchange membrane fuel cells (PEMFCs). In these cases, the thermal cracking setup must be modified, such as by incorporating gas separators. Furthermore, unlike metal annealing, PEMFCs operate at relatively low temperatures. Therefore, it is also necessary to design catalysts with high conversion at low temperatures.

Mechanistic understanding is fundamental to catalyst development. As shown in Equation 1, adsorbed NH_3_ experiences successive N‐H bond cleavage and forms H atoms. Subsequently, these H atoms couple together and generate H_2_. Finally, adsorbed N atoms form N_2_ and desorb from the catalyst surface. Based on this pathway, the rate‐determining step (RDS) of each catalyst can be confirmed, and the performance can be explained and enhanced. For example, Ganley et al. measured the turnover frequencies (TOFs) of various metals supported on Al_2_O_3_, the TOFs follow the trend: Ru > Ni > Rh > Co > Ir > Fe >> Pt > Cr > Pd > Cu >> Te [25]. Applying the Blowers–Masel equation and enthalpy data, the authors calculated the activation energies of N‐H bond scission and N‐N recombination of these metals. They observed a volcano‐shaped relationship between N‐H bond scission activation energy and the logarithm of the TOF ratio of metals to Ru. As demonstrated in Figure 2a, on the left side of the volcano, where the activation energy of N‐H bond cleavage is low, it is the N‐N coupling that controls the reaction rate. In contrast, when the activation energy of N‐H bond cleavage is high, this step limits the reaction kinetics. In particular, Ru and Ni are on the top of the volcano, indicating their activation energies are optimal for thermal cracking. More importantly, despite the relatively low performance of Ni, it exhibited 50 times lower dispersion than Ru on Al_2_O_3_, suggesting the dramatically higher intrinsic reactivity of Ni. This implies the great potential of regulating Ni dispersion to improve the performance, such as by mediating the metal‐support effect, which we will discuss later.

(1a) $$*NH_{3}\to *NH_{3-x}+x*H$$

(1b) $$2*H\to H_{2}$$

(1c) $$2*N\to N_{2}$$

(1d) $$\mathrm{Over}\mathrm{all}:\;\;2NH_{3}\;\to \;N_{2}+3H_{2}\;\Delta H^{⊖}=+92\;\mathrm{kJ}\;\mathrm{mo}l^{-1}$$

**FIGURE 2:**
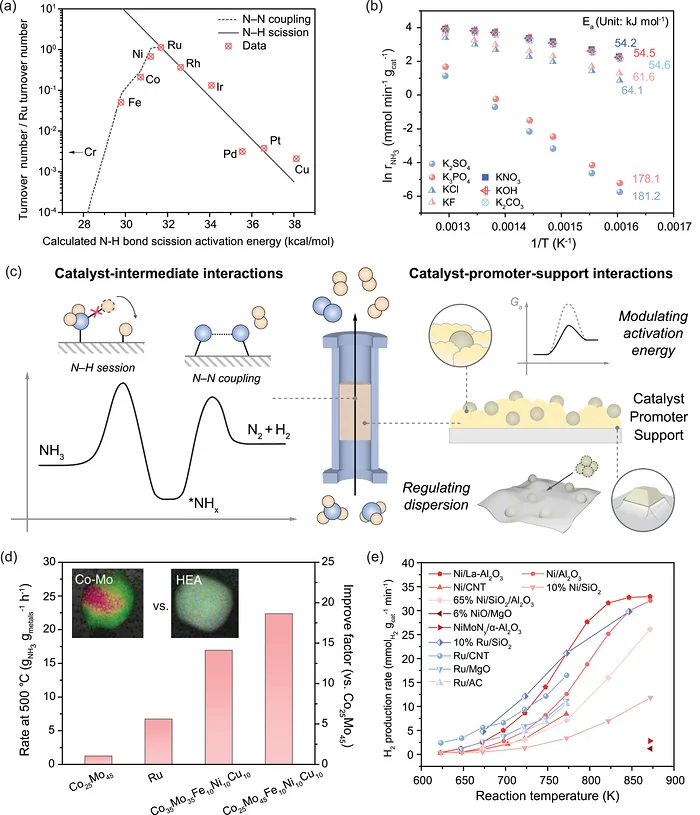
(a) Volcano plot of TOF as a function of N─H bond scission activation energy. Reproduced with permission [25]. Copyright 2004, Springer Nature. (b) Arrhenius relationship and calculated activation energies of Ru/CNT with various promoters. Reproduced with permission [28]. Copyright 2004, Elsevier. (c) Schematic illustrations of research directions of NH_3_ thermal cracking. Blue and orange balls indicate N and H, respectively. (d) NH_3_ reaction rates of Co_25_Mo_45_, Ru, Co_35_Mo_35_Fe_10_Ni_10_Cu_10_, and Co_25_Mo_45_Fe_10_Ni_10_Cu_10_ at 500°C. Inset: EDS mapping results of Co_25_Mo_45_ (Co‐Mo) and Co_25_Mo_45_Fe_10_Ni_10_Cu_10_ (HEA) nanoparticles. Reproduced under terms of the CC‐BY license [30]. Copyright 2019, The Authors, published by Springer Nature. (e) H_2_ production rates of Ni‐based and Ru‐based catalysts. Reproduced with permission [29]. Copyright 2005, Elsevier.

In addition to focusing on catalyst‐intermediate interactions for intrinsic performance, the interplays among active phases, promoters, and supports have been explored to further enhance the overall cracking performance of catalysts. (Figure 2c) Several studies have reported the improved performance after employing promoters, such as compounds with alkali metals or rare earth elements. For example, Itoh and co‐workers observed that after adding CeO_2_, Fe powders almost doubles NH_3_ conversion at 700 K [26]. They attributed this improvement to three aspects: more suitable NH_3_ adsorption, less Fe aggregation, and optimized Fe electronic structures. Varisli's group applied KOH as the promoter in SiO_2_‐supported Ru. After incorporation, under the conditions of 600°C and a gas hourly space velocity (GHSV) of 3.6×10^5^ mL g_cat_^−1^ h^−1^, the NH_3_ conversion increased drastically from 23% to 73% [27]. BET results indicated that adding KOH slightly decreases the surface area, implying that KOH modifies the electronic structure of Ru and alters the intrinsic reactivity. A comprehensive study by Wang et al. also supported this conclusion [28]. They added a wide range of alkaline metal salts as promoters in carbon nanotube supported Ru (Ru/CNT) and observed that promoters reduced BET area but negligibly changed the particle size and morphology of Ru. Regarding the performance, the authors calculated the activation energies based on Arrhenius relationship. As shown in Figure 2b, the effect of anions varied: SO_4_^2−^ and PO_4_^2−^ dramatically elevate the reaction barrier, whilst NO_3_^–^, OH^−^, CO_3_^2−^, F^−^, and Cl^−^ decrease it. On the contrary, all cations reduce the activation energy and follow the order of K > Na > Li > Ce > Ba > La > Ca. In terms of Ni, rare‐earth metal and oxides have been proven more efficient promoters compared to alkaline metal chemicals, such as KOH [23]. Xu's group demonstrated that after incorporating La as the promoter (La:Ni = 0.22), the NH_3_ conversion escalated from 38.2% to 63.9% at 500°C [29]. More importantly, at 550°C, Ni_1.20_La_0.22_/Al_2_O_3_ remained a NH_3_ conversion of 96.9% at a high GHSV of 3×10^5 ^mL g_cat_^−1^ h^−1^, and the catalyst exhibited outstanding H_2_ production rate, surpassing other candidates (Figure 2e). The group dived into the role of La and ascribed the enhancement to three key aspects. First, La reduces the particle size of Ni to an optimal range of 1.8‐2.9 nm [31]. Second, temperature‐programmed desorption (TPD) and temperature‐programmed surface reaction (TPSR) data showed that the sorption behavior was optimized by La, suggesting the modification of Ni's electronic structure. Third, La disrupted the metastable Ni‐Al oxide states near the interfaces. The authors employed temperature‐programmed reduction (TPR) to measure the phase composition. They detected that La induced more Ni‐enriched phases, such as 𝛽_1_, while decreasing Ni content in Al‐enriched phases (𝛽_2_‐NiO), facilitating the phase transition from 𝛽_2_ to 𝛾 [29]. As Ni content increases, the phase is more reducible [32]. Therefore, more Ni^0^ species will form during thermal cracking, leading to the enhancement of catalytic performance.

The above study also underscores the important role of support materials for NH_3_ thermal cracking, i.e., new Ni‐Al phases near the interface were found influential to the performance. The interactions between catalysts and support materials exert complex impact on the performance. Apart from forming new phases, support materials can modify the electronic structure of catalysts and regulate particle size and dispersion of catalysts [33, 34, 35]. In addition, physical properties of support materials are also important, including surface area, electrical conductivity, and thermal stability. A wide range of support materials have been investigated, such as Al_2_O_3_ [36], SiO_2_ [25], CeO_2_ [37], and CNT [28]. It has been pointed out that the basicity of support materials is one of the important factors for high dispersion, promoted dehydrogenation and enhanced N‐N recombination [38, 39].

Besides, research has expanded to catalysts with diverse active sites, especially transition metal alloys and nitrides, to leverage the synergistic effects for enhanced performance. Among the candidates, Co‐Mo system exhibits superior reactivity, which is even higher than Ru at the same reaction condition. Ji et al. synthesized CoMo/γ‐Al_2_O_3_ from amine metallate with a metal content of solely 5.0 wt% and measured its NH_3_ thermal cracking reactivity [40]. The NH_3_ conversion approached 99.5% at 600°C and a GHSV of 3.6×10^5 ^mL g_cat_^−1^ h^−1^. The high performance was attributed to active Co_3_Mo_3_N. To further identify the optimal metal ratio of this compound for improved activity, Xie et al. prepared CoMoFeNiCu high entropy alloys with Co‐Mo ratios that are thermodynamically unfavorable according to the bimetallic phase diagram [30]. As shown in Figure 2d, energy‐dispersive X‐ray spectroscopy (EDS) results indicate that Mo and Co are distributed uniformly and stabilized via the high‐entropy effect, while phase segregation is observed for Co_25_Mo_45_. Regarding the reactivity, Co_25_Mo_45_Fe_10_Ni_10_Cu_10_ displayed a mass‐specific rate of 22.4 g_NH3_ g_cat_^−1^ h^−1^ at 500°C, which is 18 and 3 times higher than that of Co_25_Mo_45_ and Ru, respectively. Moreover, its activation energy is comparable to Ru‐based catalysts, demonstrating its great potential as a viable alternative to Ru. In addition to Mo‐Co system, theoretical studies have been conducted on Pt‐based monolayer bimetallic surfaces. Hansgen and co‐workers employed binding energy of nitrogen atoms to screen candidates and found that Ni‐Pt‐Pt surface demonstrated comparable binding energy to Ru [41]. As evidenced by TPD, it started to release N_2_ at 353°C during NH_3_ thermal cracking. Guo et al. continued the theoretical work by investigating facet effects in Pt‐based nanoparticles to evaluate TOFs and further explain the high reactivity of the catalysts [42]. However, to the best of our knowledge, experimental works validating these findings have yet to be reported.

Engineering improvements have also been pursued for thermal cracking [43]. To ensure high‐purity H_2_ and high gas separating efficiency, techniques including pressure swing adsorption, cryogenic separation, and ion‐exchange zeolites have been developed [44]. In addition, it has been proposed to burn retentate gases for heat supply, given that NH_3_ decomposition is endothermic. Yun and colleagues demonstrated that adding a burner unit increased energy efficiency from 66.46% to 72.75% [45]. Moreover, in recent years, advanced technologies, such as magnetic induction [46], Joule heating [47], and plasma [48], have been explored for energy supply in thermal cracking. However, these innovative approaches are still in their infancy and require further development to achieve practical application.

### 2.2 NH_3_ E‐cracking

NH_3_ e‐cracking involves HER and AOR in an electrolysis cell. The reactions and their standard electrode potentials vs. the standard hydrogen electrode (SHE) are shown in Equation 2. Ideally, the required potential to initiate H_2_ production is only 0.06 V, significantly lower than that of water electrolysis (1.23 V), underscoring the great potential of NH_3_ as a H_2_ carrier [49]. Besides, e‐cracking mostly operates in the alkaline conditions, since OH^–^ is crucial for dehydrogenation processes and the high catalytic reactivity [50]. Regarding catalysts for e‐cracking, Pt is employed at the cathode because of its outstanding performance in HER [51, 52]. In contrast, AOR catalysts are less developed. Currently, two main types of candidates have been studied, namely noble metal systems and Ni‐based materials, and they primarily operate at different potential regions (Figure 3a).

(2a) $$\begin{array}{cc} & \mathrm{Cath}\mathrm{ode}:\;6H_{2}O\;+\;6e^{-}\;\to \;3H_{2}+6OH^{-}\;\\ & E^{⊖}=-0.83\;V\;\mathrm{vs}\;\;\mathrm{SHE} \end{array}$$

(2b) $$\begin{array}{cc} & \mathrm{Anode}:\;2NH_{3}\;+\;6OH^{-}\;\to \;N_{2}\;+\;6e^{-}\;+\;6H_{2}O\;\\ & E^{⊖}=-0.77\;V\;\;\mathrm{vs}\;\;\mathrm{SHE} \end{array}$$

(2c) $$\mathrm{Over}\mathrm{all}:\;2NH_{3}\;\to \;N_{2}\;+\;3H_{2}\;E_{\mathrm{cell}\;}=\;0.06\;V$$

**FIGURE 3:**
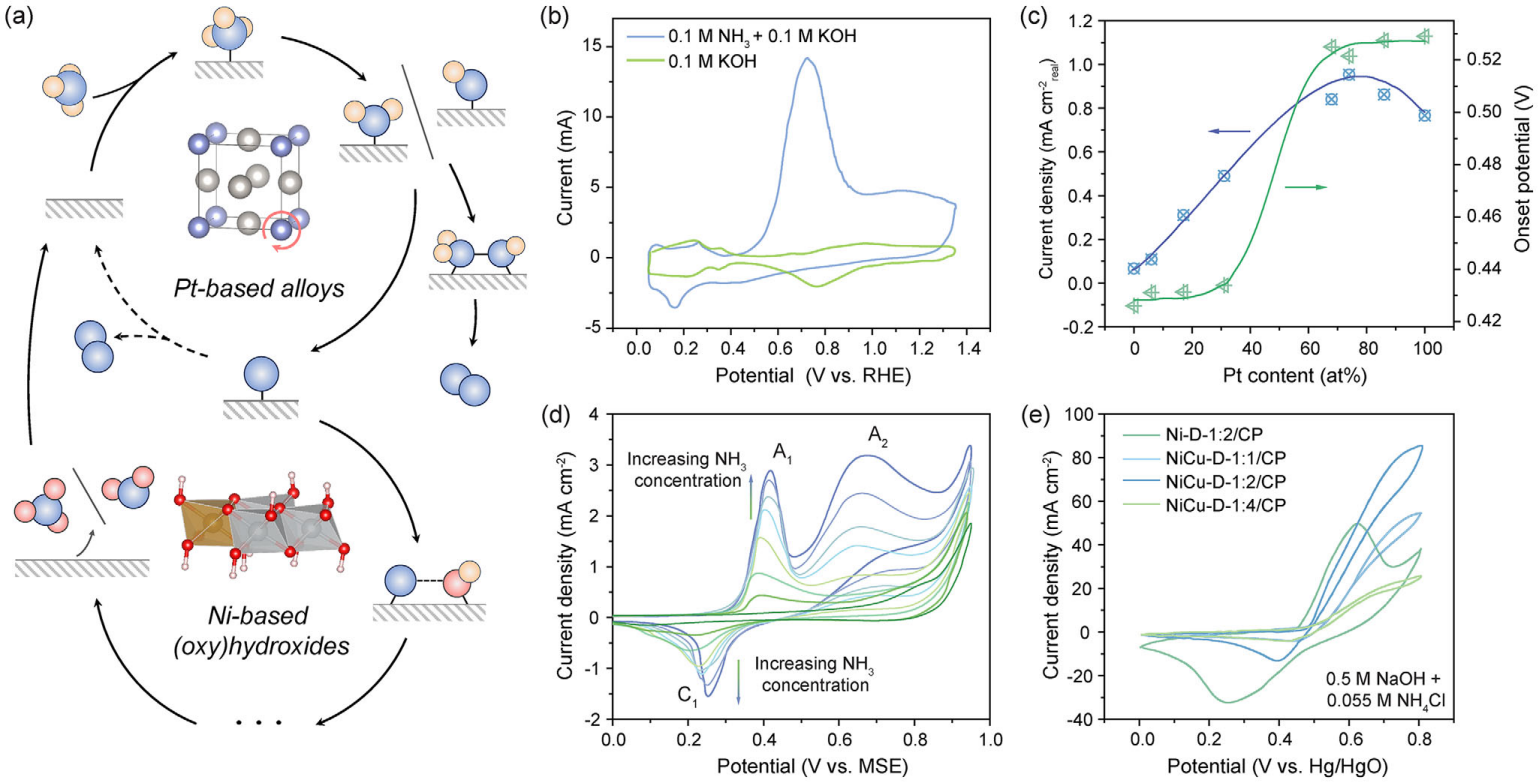
(a) Catalytic cycles of AOR on Pt‐based alloy surfaces and Ni‐based (oxy)hydroxides. Blue, red, and orange balls indicate N, O, and H, respectively. (b) Cyclic voltammograms of Pt in 0.1 m KOH with and without 0.1 m NH_3_. Reproduced with permission [53]. Copyright 1996, Elsevier. (c) AOR current densities at 0.71 V vs. RHE and onset potentials of Pt‐Ir (100) films in 1 m KOH and 0.1 m NH_3_. Reproduced with permission [54]. Copyright 2018, American Chemical Society. (d) CV curves for Ni/Ni(OH)_2_ in 1 m NaClO_4_ +NaOH at pH = 9 after adding various concentrations of NH_3_. Reproduced with permission [55]. Copyright 2009, Elsevier. (e) AOR performance of Ni and NiCu‐based catalysts with different ratios of metal precursors and dodecylamine (D) in 0.5 m NaOH and 0.055 m NH_4_Cl. Reproduced with permission [56]. Copyright 2023, American Chemical Society.

Among noble metals, Pt demonstrates the highest AOR reactivity, with both high current density and low onset potential [57, 58]. A typical AOR cyclic voltammogram on Pt is presented in Figure 3b, where current density plunges at approximately 0.7 V vs. reversible hydrogen electrode (RHE). The phenomenon is acknowledged as surface poisoning and is attributed to strong adsorption of N‐containing species. Two mechanisms were postulated to describe the detailed reaction pathway of AOR, namely Oswin−Salomon mechanism and Gerischer−Mauerer mechanism (Figure 3a). In Oswin−Salomon mechanism, NH_3_ is deprotonated to *N (* stands for an active site) through proton‐coupled electron transfer (PCET) steps, and *N then dimerizes to generate N_2_ (Equation 3a‐e). Conversely, Gerischer and Mauerer proposed that N‐N coupling occurs before the formation of *N, and the resulting *N_2_H_x+y_ then experiences several PCET steps to produce N_2_ (Equation 3a‐d, f, g), while *N poisons the surface. Given the strong poisoning on Pt during AOR, Gerischer−Mauerer mechanism is considered as the primary pathway of N_2_ production on noble metals, which is also supported by theoretical works [59].

(3a) $$*+NH_{3}\left(aq\right)\to *NH_{3}$$

(3b) $$*NH_{3}+OH^{-}\to *NH_{2}+H_{2}O+e^{-}$$

(3c) $$*NH_{2}+OH^{-}\to *NH+H_{2}O+e^{-}$$

(3d) $$*NH+OH^{-}\to *N+H_{2}O+e^{-}$$

(3e) $$2*N\to *N_{2}$$

(3f) $$*NH_{x}+*NH_{y}\to *N_{2}H_{x+y}$$

(3g) $$\begin{array}{c} {}*N_{2}H_{x+y}+\left(x+y\right)OH^{-}\to *N_{2}+\left(x+y\right)H_{2}O+\left(x+y\right)e^{-} \end{array}$$

Further studies on Pt‐based catalysts have been conducted to achieve high current density and low peak potential for higher efficiency of e‐cracking. Among these, Pt alloys, especially Pt‐Ir alloys, have garnered much attention, as Ir is the second active noble metal for AOR. Guay's group synthesized a series of Pt‐Ir alloys with varying Ir contents and measured AOR performance in 1 m KOH with 0.1 m NH_3_ [54]. As demonstrated in Figure 3c, the onset potential gradually declines with the increase of Ir content, which can be explained by the low onset potential of metallic Ir. Notably, Pt_76_Ir_24_ exhibits an electrochemically active surface area (ECSA)‐normalized current density of around 1.0 mA cm^−2^, exceeding Pt by 22%. This indicates the high intrinsic activity of the alloy due to the synergistic effects. Moreover, alloying both transition metals and noble metal with Pt offers new opportunities for AOR catalysis, as these alloys not only exhibit promising reactivity, but also substantially reduce material costs [60, 61, 62]. For example, Li et al. deposited PtIrZn_2_ on SiO_2_‐CNT‐COOH (CNT‐COOH: carboxyl‐functionalized carbon nanotube) and observed a comparable peak mass activity to PtIr, accompanied by a lower peak potential [60].

The vast compositional space of multi‐element Pt‐based alloys complicates the screening of robust AOR catalysts. Activity descriptors have thus been developed to simplify this process. Given the poisoning effect of *N, its adsorption energy was employed to explain and predict the activity [57]. According to the Sabatier principle, the intermediate binding should be neither too strong nor too weak to maximize catalytic activity [63]. Koper et al. compared the adsorption energies across noble metal surfaces and demonstrated that Pt exhibits optimal binding for both NH_3_ dehydrogenation and N‐N coupling, a balance that underlies its superior activity [57]. Moreover, Xin's group correlated the binding energies of bridge *N and hollow *N with those of the other AOR intermediates and constructed a microkinetic model for evaluating the turnover frequency (TOF) toward N_2_ [64]. After screening over 20,000 candidates, they identified Pt_3_Ru_0.5_Co_0.5_ as the most active Ir‐free Pt‐based catalyst. In addition to binding energies, the upper d‑band edge energy of the metal site (ε_u_) has been employed as an electronic descriptor for AOR performance [65]. Since alloying can substantially distort the d‑band shape, ε_u_ has been shown to capture activity trends more reliably than the conventional d‑band center in these systems. Fang et al. reported that the AOR activity of Pt‐Co alloys exhibits a volcano‐type dependence on composition, with Pt_7_Co_2_ displaying the lowest limiting potential. Notably, this trend was linearly correlated with the ε_u_ change (R^2^ ≈ 0.90) and also validated by electrochemical measurements.

Beyond the computational descriptors, oxophilicity has been proposed as an experimentally accessible descriptor for the onset potential [66]. CO‐stripping voltammetry and in situ ATR‐FTIR spectra indicate that oxophilic surfaces tend to adsorb *OH at lower potentials, which facilitates NH_3_ dehydrogenation and thus determines the onset of AOR. This is also consistent with the theoretical results [67], where the dehydrogenation by *O/*OH was shown to dominate over that by free OH^–^ in the electrolyte. Other studies, however, suggest that these species may poison the surface. For instance, Zhuang et al. observed sustained AOR on Pt in non‐aqueous CH_3_CN [68]. As both aqueous and non‐aqueous AOR share the *N intermediate, this implies that *N is not the sole poisoning species. Furthermore, Perez's group reported that pre‐oxidation at 0.9 V vs. RHE in 1 M NaOH markedly suppressed the AOR current density of Pt, whereas pre‐oxidation at 0.7 V vs. RHE left the AOR profile essentially unchanged [69]. Collectively, another volcano relationship regarding oxophilicity can be formulated from these findings. Specifically, weakly oxophilic surfaces are unfavorable for *O/*OH adsorption and the subsequent dehydrogenation, whereas strongly oxophilic surfaces are readily oxidized under operating potentials and thus deactivated, as exemplified by Ru/RuO_x_ [70]. Identifying compositions at the top of both volcano relationships therefore remains a challenge. By exploiting the compositional variability of Pt‐based alloys together with these two descriptors, catalysts can be rationally designed to resist poisoning.

Regardless of the precise identity of the poisoning species, the catalyst can be recovered by briefly applying a low potential [71, 72]. Therefore, engineering strategies utilizing this reversibility have been adopted to sustain e‐cracking operation despite surface poisoning. For example, Shin et al. validated this concept by comparing two operating protocols in the same Zirfon‐separator cell, which delivered a peak current density of 915 mA cm^−2^ at 90°C in 8 M KOH and 1 M NH_3_ [73]. Under continuous CA at 80°C and 0.6 V vs. RHE, the cell deactivated completely within 12 h, whereas applying 0.0 V vs. RHE for 1 min after every 2 min of AOR operation retained 56% of the initial current density after 100 h. Xu et al. further developed an electrode alternating strategy for stable H_2_ production [74]. The periodic exchange of the cathode and anode enabled the e‐cracking membrane electrode assembly (MEA) prototype to sustain a current density of approximately 2 A cm^−2^ for 1,000 h in 1 m KOH and 3 m NH_3_. This approach, termed polarity‐switching by Botte and colleagues, was applied in a 3,000 cm^2^ zero‐gap bipolar pilot‐scale e‐cracking cell [75]. After 58 h of operation, the current density declined by only 20%. These examples underscore the viability of this dynamic regeneration approach for scale‐up, an essential step toward practical implementation of e‐cracking systems.

Another category of AOR catalyst is Ni‐based materials, which have displayed superior performance in other electrochemical oxidation reactions, such as methanol oxidation [76] and urea degradation [77]. In AOR, these catalysts can oxidize the poisoning species to mitigate activity drop, but they typically require a relatively high operating potential. A representative study was conducted by Kapałka et al., who measured cyclic voltammetry (CV) curves on pre‐oxidized Ni in solutions with varying NH_3_ concentrations. As depicted in Figure 3d, two anodic peaks and one cathodic peak were detected in the voltammograms. Specifically, Peak A1 occurs at ca. 1.47 V vs. RHE (0.3 V vs. Hg/Hg_2_SO_4_/K_2_SO_4_, MSE), which should be ascribed to the oxidation of Ni(II) to Ni(III) [55]. Since A1 shows NH_3_ dependence, the authors hypothesized an NH_3_‐containing intermediate, denoted as NiOOH(*NH_3_) for the redox process at A1. This compound is subsequently oxidized to release N_2_, which contributes to peak A2 (Equation 4). The cathodic peak C1 was then attributed to the reduction of NiOOH and unreacted NiOOH(*NH_3_), as C1 exhibited less NH_3_ dependence. The authors also performed chronoamperometry (CA) and found that current density did not gradually decline. More importantly, nitrate was detected by a spectrophotometer, suggesting the catalyst's capability of oxidizing poisoning species like *N to maintain its reactivity.

(4a) $$Ni{\left(OH\right)}_{2}+NH_{3}+OH^{-}\to NiOOH\left(*NH_{3}\right)+H_{2}O+e^{-}$$

(4b) $$\mathrm{NiOOH}\left(*NH_{3}\right)+OH^{-}\to \mathrm{NiOOH}+N_{2}+e^{-}$$

The oxidation of poisoning species generates other products in addition to N_2_, including NO_2_^–^ and NO_3_^–^. Regarding selectivity, the detailed mechanism remains elusive, and two relevant factors have been explored. First, some studies correlate the generation of high‐valent Ni^III^ sites with NO_x_ formation. Phan and colleagues employed in situ attenuated total reflection‐surface‐enhanced infrared absorption spectroscopy (ATR‐SEIRAS) and demonstrated that the formation potential of NO_3_^–^ coincided with the phase transition from Ni(OH)_2_ to NiOOH [78]. Moreover, OH/O vacancies are proposed as active sites for NH_3_ adsorption and subsequent oxidation. However, due to surface reconstruction at high potentials, the precise correlation between these vacancies and selectivity remains unquantified.

The second factor is the synergistic effect between Ni and a secondary metal dopant. Cu is one of the most studied elements, and its incorporation has been shown to enhance N_2_ selectivity and overall current density. For instance, Vu and colleagues employed a solvothermal method to synthesize Ni and NiCu catalysts using dodecylamine as a precipitation agent, denoted as Ni‐D and NiCu‐D, respectively [56]. As shown in Figure 3e, NiCu‐D/CP (CP: carbon paper) achieves a current density of approximately 90 mA cm^−2^ at 1.62 V vs. RHE (0.7 V vs. Hg/HgO) in 0.5 M NaOH and 0.055 m NH_4_Cl, higher than that of Ni‐D. These results indicate the synergistic effects between Ni and Cu. Theoretical calculations further indicate that Cu facilitates N─N coupling through the Gerischer−Mauerer mechanism and hinders the over‐formation of poisoning *N by Ni phases. In contrast, when Cu is absent, *N coverage can easily reach a high level, and *N must be oxidized to NO_x_ to recover the catalyst surface. This hypothesis was further supported by the Faradaic efficiency results, where the main AOR product of NiCu‐D/CP is N_2_, while high NO_x_ concentration was detected for Ni‐D/CP. Beyond Cu, a study on AOR performance over monometallic (oxy)hydroxides indicated that Fe operates through an O_2_‐dependent mechanism, as the product distributions are significantly different in Ar‐saturated and O_2_‐saturated electrolytes [79]. Fe in NiFe (oxy)hydroxides may therefore also regulate reaction pathways. Since the operating potential of the AOR overlaps with that of the oxygen evolution reaction (OER), quantifying the evolved O_2_, for instance by differential electrochemical mass spectrometry, is necessary to isolate the AOR contribution when mechanisms are examined. Interestingly, the O_2_‐dependence observed on Fe suggests that O_2_ may act as a co‐reactant rather than merely a competing product, coupling the OER to the AOR pathway.

Benefiting from the mitigated poisoning, researchers can evaluate cell performance for NH_3_ e‐cracking. Graves et al. assembled a flow cell anion exchange membrane ammonia electrolyzer (AEMAE) using NiCu/NF (NF: nickel felt) and Pt/C as the anode and cathode, separately [80]. The cell managed to run over 20 days with 100 mA cm^−2^ current density and a cell voltage of 1.5 V in 1 m KOH and 1 NH_3_. The catalyst demonstrated high selectivity for both N_2_ and H_2_, with Faradaic efficiencies achieving 88% and 99%, respectively. These results provide implications on the great potential of Ni‐Cu (oxy)hydroxides for NH_3_ e‐cracking, while the performance improvements remain necessary to meet practical demands.

Regardless of current limitations in catalyst development, we would like to emphasize several advantages of e‐cracking over thermal cracking. First, e‐cracking drastically reduces extra energy costs for sustaining the reaction. Although both techniques necessitate energy input due to the endothermic nature of NH_3_ decomposition, the setup of thermal cracking imposes a higher extra energy demand. During the process, NH_3_ must be preheated before entering the reactor to maintain the reaction temperature. When it flows out, thermal energy was carried away from the reactor, causing energy loss. Moreover, gas separator is necessary for thermal cracking, especially for applications demanding high‐purity H_2_, which further lowers energy efficiency. For example, a typical pressure swing adsorption (PSA) can recover only 75% of H_2_ from the mixed gas [81]. Hence, more H_2_ must be produced to fulfill daily production requirement. Conversely, e‐cracking generates gases at different electrodes with negligible thermal energy input and associated losses, leading to a higher energy efficiency. We quantitatively calculated energy efficiencies of two cracking methods (Figure 4). For e‐cracking, this is defined as the LHV of the hydrogen produced divided by the total energy input, comprising the LHV of the ammonia consumed and the electrical energy. Details of energy efficiency calculation are elucidated in Supporting Information. For thermal cracking, we adopted the results from Yun et al. [45]. and focused on two reactor configurations: a base model (base) and one equipped with a burner to combust retentate for heat supply (burner). The corresponding energy efficiencies, based on the H_2_ flow rate at the outlet of the PSA units, were estimated as 66.46% and 72.75%. As illustrated in Figure 4, the energy efficiency of e‐cracking decreases when increasing cell voltage. Calculation results suggest that e‐cracking has a higher energy efficiency than thermal cracking without and with a burner when voltage is below 0.80 V and 0.63 V, respectively.

**FIGURE 4:**
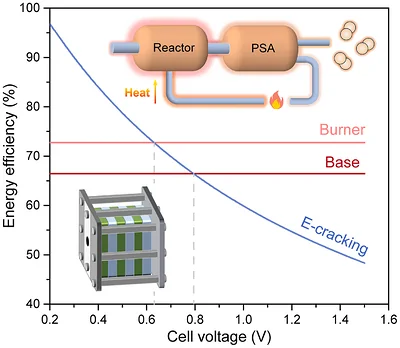
Energy efficiencies of e‐cracking, thermal cracking base model, and thermal cracking model with a burner.

Second, e‐cracking enables NH_3_ decomposition in decentralized scales. Unlike thermal cracking that needs complex and large‐scale units, electrolysis cells for e‐cracking have much smaller, more compact setups, which are more convenient and suitable for off‐grid applications. Furthermore, given that reactant is an NH_3_ aqueous solution rather than gaseous NH_3_, ammonium salts can be utilized in e‐cracking, which further reduces the cost and size requirement for storage units. These advantages underline the promising prospects of NH_3_ e‐cracking as a sustainable and scalable approach for H_2_ production.

## 3 Techno‐Economic Analysis

To quantitatively evaluate the technical and economic viability of NH_3_ e‐cracking, we conducted TEA to calculate LCOH assuming a daily H_2_ production of 1,500 kg, following hydrogen analysis (H2A) hydrogen production models [82]. The overall LCOH comprises two components: capital expenditure (CAPEX) and operational expense (OPEX). Details of LCOH calculation methodology are provided in Supporting Information. Considering the differences in costs and performance between Pt‐based and Ni‐based catalysts, we assessed the LCOH of three cases, where AOR is catalyzed by Pt_9_Ir, PtIr, and NiCu/NF. Table 1 summarizes the input data for TEA, where performance is adopted from the literature and price of catalysts is calculated using market data [83]. Regarding catalyst durability, two distinct forms of activity loss were treated in TEA, namely surface poisoning and catalyst degradation. Although severe surface poisoning can result in a remarkable current density drop in CV and CA measurements, as mentioned in Section 2.2, the activity has been shown to be restored by applying a low potential. Botte et al. proposed that applying brief cathodic pulses could efficiently remediate surface activity. They determined the optimal durations of 120 s and 1 s for the anodic step and the cathodic step, respectively [71]. Consequently, we incorporated this approach into our calculations for Pt_9_Ir by decreasing daily H_2_ production to compensate for the time allocated to the poisoning recovery procedure. Moreover, we considered an ideal catalyst, denoted as Pt_9_Ir‐ideal, which can overcome surface poisoning and exhibit considerable current density. The performance data were adopted from the cell polarization curve, where the poisoning effects have limited influence on it. As for catalyst degradation, catalyst replacement should be considered. However, this would necessitate accounting for precious element recycling. While the recycling process potentially reduces net catalyst cost, it introduces additional uncertainties in overall cost estimation [84]. Therefore, catalyst replacement is incorporated into the cell compartment replacement term in this study.

**TABLE 1:** Input data of catalysts for LCOH calculations.

Catalyst	Current Density [mA cm^−2^]	Voltage [V]	Reference	Price [$ g^−1^]
Pt_9_Ir	80	0.55	[71]	44.30
Pt_9_Ir‐ideal	300	0.80	[71]	44.30
PtIr	100	0.40	[85]	93.70
NiCu/NF	125	1.70	[80]	0.013

As demonstrated in Figure 5a, the calculated LCOH for Pt_9_Ir, PtIr, and NiCu/NF are $4.71 kg_H2_^−1^, $4.99 kg_H2_^−1^, and $4.64 kg_H2_^−1^. Assuming an NH_3_ spot cost of $500 ton^−1^, feedstock cost is $2.81 kg_H2_^−1^ and represents the dominant component of the overall LCOH for all catalysts. Furthermore, the annualized CAPEX constitutes the second‐largest contributor for noble metal catalysts, consistent with their high catalyst price and the associated stack costs. Notably, while the annualized CAPEX for NiCu/NF is relatively low, electricity cost ($1.35 kg_H2_^−1^) is remarkably higher than that of other catalysts, due to its high operating potential. Despite this, NiCu/NF achieves the lowest overall LCOH among three real catalysts, at $4.64 kg_H2_^−1^. Regarding Pt_9_Ir‐ideal, LCOH is only $3.87 kg_H2_^−1^, with feedstock being the largest contributor. In other words, e‐cracking costs approximately $1 kg_H2_^−1^, highlighting its significant economic value for H_2_ recovery.

**FIGURE 5:**
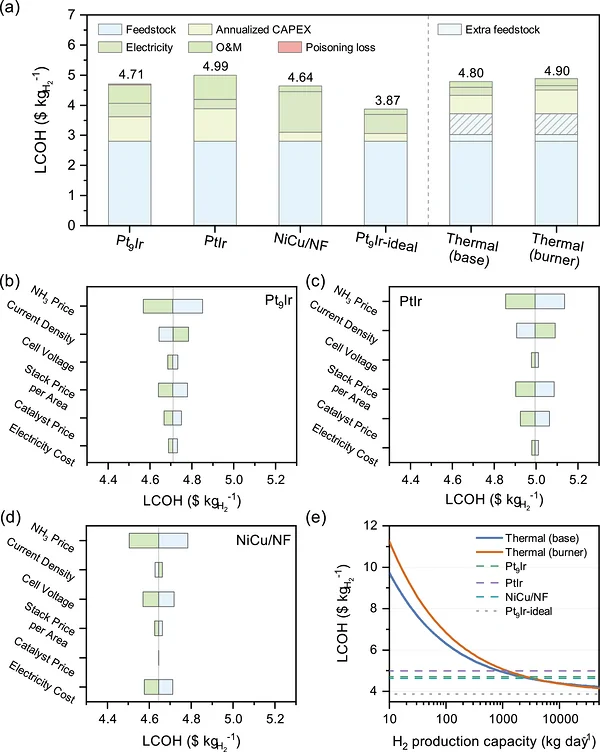
(a) LCOH of e‐cracking with Pt‐based and Ni‐based AOR catalysts, and thermal cracking. For thermal cracking, LCOH reduction by optimizing PSA efficiency is represented as the shaded area. O&M comprises maintenance and stack replacement for e‐cracking, and maintenance, operating supplies and cooling water for thermal cracking. (b‐d) Sensitivity analysis of (b) Pt_9_Ir, (c) PtIr, and (d) NiCu/NF. (e) LCOH for both cracking techniques as a function of H_2_ production capacity. Details of the LCOH calculation are provided in Supporting Information.

To further illustrate the contributions of each component to LCOH, we conducted sensitivity analysis, where each factor is varied by ±5% to assess the change in LCOH. The results are shown in Figure 5b–d, where green and blue represent the changes when alter +5% and −5% of each component, respectively. All three cases demonstrate high sensitivity to NH_3_ price, which leads to a fluctuation of $0.14 in LCOH. In addition, LCOH of noble metal catalysts is highly sensitive to stack cost per area and current density, and the price fluctuation is positively correlated with Ir content. Besides, changes electricity price and cell voltage have less impact on LCOH (around $0.02). Alternatively, the LCOH of NiCu/NF exhibits higher dependence on cell voltage and electricity price, both of which result in approximately $0.07 alteration. Whereas the price fluctuation caused by current density and stack costs are only $0.02, indicating that reducing cell voltage is more crucial than increasing current density for Ni‐Cu hydroxides regarding economic value.

We also calculated LCOH for NH_3_ thermal cracking, and the performance data were adopted from Lee et al. [86]. They fed NH_3_ at a flow rate of 5.88 kmol/h into the reactor and obtained 4.23 kmol/h H_2_ after gas separation via PSA. Details of the LCOH calculation for thermal cracking are provided in Supporting Information, and the production rate was also scaled to a comparable basis (1,510 kg day^−1^), with equipment cost estimated using the six‐tenths rule. As illustrated in Figure 5a, the calculated LCOH for base model and burner model is $4.80 and $4.90 kg_H2_^−1^, respectively. Notably, to ensure high H_2_ purity, the PSA recovery ratio is only 79%, necessitating additional NH_3_ feedstock and elevating overall LCOH. We also considered an improved PSA process with a 5% increase in recovery efficiency, as presented in Figure 5a. The enhancement substantially declines the feedstock cost by $0.30, indicating the essential roles of PSA efficiency and NH_3_ spot price in determining LCOH. Previous studies have suggested that a lower H_2_ purity requirement allows higher efficiency [87, 88], indicating that LCOH for thermal cracking can be further reduced under specific scenarios. For example, Ammonigy has proposed an interim solution that bypasses purification altogether by directly combusting the NH_3_‐H_2_ mixture. They replaced the diesel engine of a speedboat with a NH_3_‐fueled gas turbine with a NH_3_ thermal cracking unit in 2022. The overall power output of the system fulfills the requirement of low‐speed test (< 10 km h^−1^), and following scale‐up calculations indicated that only twice the size of the installed cracker is needed for a 100‐meter‐long vessel carrying 1,000 ton of freight [89]. In such scenarios, the PSA can be waived, and the feedstock will be reduced by 20% ($2.93 kg_H2_^−1^), resulting in a much lower LCOH of $4.00 kg_H2_^−1^.

To further distinguish decentralized scenarios from centralized ones, we calculated the dependence of LCOH on H_2_ production capacity (Figure 5e). Following the six‐tenths rule, the LCOH of thermal cracking rises steeply as capacity decreases. Notably, a 300‐kW semi‐truck corresponds to a hydrogen demand of ∼9 kg h^−1^ (∼216 kg day^−1^) on an energy‐equivalent (LHV) basis, and higher once conversion losses are included. The corresponding LCOH of thermal cracking reaches $6 kg_H2_^−1^. In contrast, the LCOH for e‐cracking remains unchanged, as the electrolyzer is assembled from repeating modular stacks whose cost scales linearly with capacity. The two routes intersect at approximately 2,000–2,800 kg day^−1^ depending on the anode catalyst, equivalent to a hydrogen output of 3–4 MW on an LHV basis. Below this range e‐cracking is the cheaper option, whereas above it thermal cracking retains the advantage. As our sensitivity analysis suggests catalytic performance is the second most important factor in decreasing cost, this intersection is largely affected by catalyst performance. Consequently, we further predicted and plotted heatmaps of LCOH vs. cell voltage and current density for Pt_9_Ir and NiCu/NF to evaluate the economic potential of e‐cracking (Figure 6). Here, a target price of $3.90 kg_H2_^−1^ was selected for e‐cracking, since the treatment cost, which is calculated as the LCOH minus feedstock cost, is comparable to that of thermal cracking, approximately $1.10 kg_H2_^−1^.

**FIGURE 6:**
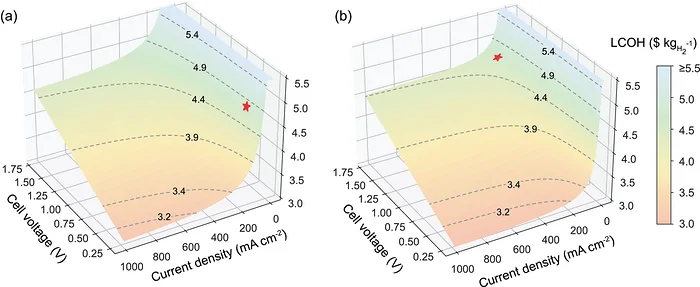
Heatmaps of predicted LCOH vs. cell voltage and current density for (a) Pt_9_Ir and (b) NiCu/NF catalysts. Red stars indicate reported performance. (Pt_9_Ir: 80 mA cm^−2^, 0.55 V; NiCu/NF: 125 mA cm^−2^, 1.70 V).

As shown in Figure 6a, increasing current density drives down LCOH more significantly in low potential regions, and achieving $3.90 kg_H2_^−1^ is almost infeasible when cell voltage exceeds 1.15 V. Moreover, based on our energy efficiency results, cell voltages of 0.63 and 0.80 V are critical thresholds for achieving higher energy efficiency than thermal cracking with burner and base model, respectively. At these voltages, the corresponding required current densities to achieve the LCOH of $3.90 kg_H2_^−1^ are 200 and 280 mA cm^−2^, separately, which are much lower than those in water electrolysis [90]. Notably, Pt_9_Ir achieved a current density of 300 mA cm^−2^ at 0.8 V in the cell polarization curve, which we used to calculate LCOH of Pt_9_Ir‐ideal. This reflects the strongly negative impact of surface poisoning. Consequently, future research should focus on developing catalysts that follows alternative reaction pathways to take advantage of poisoning *N and mitigate activity decay. Furthermore, given the relatively low target current density, screening Pt‐based catalysts with considerable current density remains a viable strategy for operating e‐cracking with lower treatment cost than thermal cracking, even than water electrolysis.

Figure 6b presents the LCOH predictions of NiCu/NF. Owing to the much lower price of NiCu/NF, its predicted LCOH is cheaper than that of Pt_9_Ir. The contour lines indicate that once current density becomes sufficiently high, further increases have negligible impact on LCOH. In other words, LCOH exhibits low sensitivity to current density, aligning well with our sensitivity analysis. Regarding the cell voltage, since Ni‐Cu hydroxides demand high potential (> 1 V) to retain activity, comparable energy efficiency to thermal cracking is not achievable for these catalysts. We thereby selected 1.00, 1.10, and 1.20 V as targeted potentials and determined the corresponding current densities required to reach LCOH of $3.9 kg_H2_^−1^ to be 207, 333, and 1,000 mA cm^−2^. Although the capability of Ni‐Cu hydroxides to maintain activity allows them to deliver considerable current density, our findings demonstrate that decreasing cell voltage is more essential for optimizing these catalysts. Further studies should concentrate on incorporating highly oxidative species for enabling AOR at lower potentials. Besides, it is commonly acknowledged that Ni (oxy)hydroxides may produce NO_x_ during AOR, [91] where more electrons are transferred per NH_3_ molecule, implicating a reduced NH_3_ feedstock demand and lower LCOH for these catalysts. Hence, product selectivity is also an essential aspect in future research.

Given the high sensitivity of LCOH to NH_3_ spot price, we further explored its current variability and potential future trends in LCOH. In this work, we employed $500 per ton NH_3_ for LCOH calculations. According to a report by the International Renewable Energy Agency (IRENA), NH_3_ prices vary by locations and synthesis procedures [92]. If the price of $375 ton^−1^ is adopted, LCOH can be reduced by $0.70 kg_H2_^−1^. Combined with the target treatment cost of $1.10 kg_H2_^−1^, the overall LCOH would reach $3.20 kg_H2_^−1^, which is the current spot price of H_2_ [83]. This indicates the potential profitability of e‐cracking when utilizing cheaper NH_3_ sources. Rising prices, conversely, penalize thermal cracking more heavily, as its larger feedstock requirement scales with the NH_3_ price. Renewable NH_3_ produced at locations with the best solar and wind resources is currently estimated at $720 ton^−1^ [92]. At this price, all three catalysts fall below the thermal route ($6.43 kg_H2_^−1^), with LCOH values of $5.95, $6.23 and $5.88 kg_H2_^−1^ for Pt_9_Ir, PtIr and NiCu/NF, respectively. Looking further ahead, IRENA predicted that the price of green NH_3_ will decline to $310 ton^−1^ by 2050 [92], resulting in an intriguing green H_2_ price of $2.84 kg_H2_^−1^. Furthermore, the engineering merits of e‐cracking enables the usage of ammonium salts as feedstock. Currently, the spot prices of NH_4_Cl in North America and Northeast Asia are only $250 and $80 ton^−1^, separately [83], implying the further decrease in LCOH for NH_3_ e‐cracking.

## 4 Conclusion and Perspectives

H_2_ utilization is critical for the transformation from traditional fossil fuels to renewable energy sources. However, the transportation and storage of H_2_ require high‐pressure compression or low‐temperature liquefaction, posing great challenges in economics and energy efficiency for establishing a worldwide H_2_ supply chain. To address these issues, NH_3_ is employed as a H_2_ carrier. H_2_ is converted into NH_3_ for subsequent transportation, and NH_3_ is broken down to release H_2_ after arrival. Two primary methods have been proposed for NH_3_ decomposition: thermal cracking and e‐cracking. E‐cracking, as an emerging electrochemical technique, has a lower technology readiness level (TRL) than thermal cracking, and its market competitiveness remains unexplored. This perspective article reviews current catalyst development of both techniques and compares the economic feasibility of e‐cracking with that of conventional thermal cracking.

We assess these procedures regarding the energy efficiency and LCOH. Our results indicate that 0.80 and 0.63 V are two key potential thresholds to reach higher energy efficiency for e‐cracking compared to base model and burner model for thermal cracking, respectively. Moreover, TEA is conducted to illustrate the current situation and the future of these procedures. Based on the ammonia oxidation catalysts reported to date, LCOH of Pt_9_Ir, PtIr, and NiCu/NF was estimated at $4.71 kg_H2_^−1^, $4.99 kg_H2_^−1^, and $4.64 kg_H2_^−1^, separately. In addition, sensitivity analysis revealed that LCOH is highly sensitive to current density and cell voltage for Pt‐based and Ni‐based catalysts, respectively, offering valuable guidance for future catalyst design. Regarding thermal cracking, we estimated LCOH for base and burner models as $4.80 kg_H2_^−1^ and $4.90 kg_H2_^−1^, respectively. We identified that current low PSA efficiency causes an extra feedstock demand to fulfill the daily production requirements, and thereby significantly raises LCOH. Whereas the treatment cost is as low as $1.1 kg_H2_^−1^ due to the mature setup and lower CAPEX. We provide perspectives for future development of NH_3_ e‐cracking based on our findings. Future endeavors can be devoted to:

(i) Developing robust AOR catalysts for e‐cracking. The remarkable price gap between real catalysts and Pt_9_Ir‐ideal suggests the essentiality of catalyst development in AOR. Considering their distinct electrochemical behaviors, we investigated both noble metal and non‐noble metal catalysts, using Pt‐based and NiCu‐based catalysts as benchmarks. Assuming a target treatment cost of $1.10 kg_H2_^−1^, the demanded current densities of Pt_9_Ir are 200 and 280 mA cm^−2^ at 0.63 and 0.80 V, respectively. Particularly, our findings in sensitivity analysis and LCOH heatmap predictions identify the current density limitation as a primary obstacle for further LCOH reduction in noble metal catalysts. Owing to their low operating potential, the catalysts require relatively low current densities to achieve the target treatment cost. Hence, despite the incomplete elimination of surface poisoning, moderate improvement in current density can be attained in the future to achieve the target cost through alloying and doping strategies. In contrast, non‐noble catalysts, typically Ni‐Cu systems, offer lower CAPEX and inherent resistance to surface poisoning. We determine that for NiCu/NF, when the cell voltages are 1.00, 1.10, and 1.20 V, corresponding current densities demanded to reach the target treatment cost are 207, 333, and 1000 mA cm^−2^. While high current density is attainable thanks to the elimination of surface poisoning, the high cell voltage elevates electricity cost and thereby increases LCOH, which is supported by our sensitivity analysis. Therefore, further efforts can be devoted to unlocking AOR at lower potentials for these non‐noble catalysts. Additionally, further studies can also explore the economic feasibility of NH_3_ oxidation to NO_2_^–^/NO_3_^–^, which requires less feedstock and may offset the high electricity cost.
(ii) Refining the design of e‐cracking devices. Engineering strategies have been shown to address the surface poisoning in e‐cracking devices. Consequently, further efforts can be devoted to optimizing both catalyst performance and cell design. Notably, advances in other electrochemical technologies, such as the design of ion‐conducting components for solid‐state batteries, provide insights into such enhancements [93]. Additionally, NH_3_‐induced corrosion should be addressed. During e‐cracking, some pure metals can be oxidized and form complexes with NH_3_, such as [Pt(NH_3_)_4_]^2+^ [94], which compromises device durability. Moreover, analogous to water electrolysis, increasing the operating temperature can effectively enhance kinetics and H_2_ production. Nevertheless, the rapid generation of *N species may aggravate surface poisoning and thereby hinders the reaction. Therefore, optimization in operating conditions, including electrolyte concentration and temperature is necessary for balancing performance and stability. In addition, product management is another essential aspect. Since N_2_ and H_2_ are generated at different electrodes, the use of gas separators or membranes may be unnecessary, as demonstrated in membrane‐free water electrolyzers [95, 96]. This can further decrease device cost. Depending on the NH_3_ concentration in the electrolyte, a simple water scrubber may be required to remove residual NH_3_ contamination in H_2_. Moreover, the byproduct management, such as NO and NO_2_, may be required, depending on catalyst performance.
(iii) Addressing system integration challenges for NH_3_‐based energy systems. Beyond optimizing individual electrochemical cells, bridging the gap to practical applications requires solving complex system‐level integration issues. For example, when e‐cracking devices are integrated with PEMFCs in NH_3_ e‐cracking vehicles, their energy distribution architecture remains underexplored. System startup requires an auxiliary battery to power the electrolyzer for initial H_2_ generation, and during operation, electric power for the NH_3_ electrolyzers must be dynamically allocated from either the PEMFC or the battery to match the load. As an early demonstration, Botte et al. designed a fuel‐cell vehicle that utilizes the electricity output of the PEMFC to power both the on‐board ammonia electrolysis unit and the electric motor [97]. This design incorporates an H_2_ buffer tank to supply initial hydrogen for cold starts and to stabilize the feed during transient load changes. Notably, while conceptually insightful for vehicular integration, its physical implementation relies heavily on balancing interdependent parameters, such as power distribution, battery capacity, and H_2_ flow rates. Establishing rigorous theoretical frameworks and system‐level design standards is thus imperative to realize practical demonstrations of these integrated systems in the future.
(iv) Establishing NH_3_ cracking routes by integrating complementary approaches. We have demonstrated the advantages of e‐cracking in flexible and decentralized applications, whereas thermal cracking is more suitable for large‐scale industrial H_2_ production. Instead of treating them as competing technologies, a complementary strategy that integrates both NH_3_ cracking systems could maximize the benefits of NH_3_ as a hydrogen carrier. Moreover, in decentralized scenarios, since both routes deliver H_2_ into the same downstream architecture, they encounter a largely common set of integration challenges. Engineering precedents established by the more mature route are therefore transferable to the emerging one. Owing to the higher TRL of thermal cracking, several prototypes have been developed combining this technique with PEMFCs. For instance, Amogy validated its ammonia‐to‐power platform in a 5‐kW drone in 2021 and a 300‐kW semi truck in 2023, with both utilizing onboard NH_3_ thermal cracking [98]. While the maturity of thermal cracking facilitates early integration into energy systems, its high operating temperature and downstream purification requirements intrinsically complicate the transition from prototype to practical deployment. In this regard, thermal cracking offers both a near‐term route and a design reference for e‐cracking systems. As its catalytic performance advances, e‐cracking can in turn relieve these constraints through a more compact setup. Coordinating the two routes across deployment scale and technological maturity could therefore accelerate the practical use of NH_3_ as a hydrogen carrier.
(v) Advancing NH_3_ synthesis for greener and cheaper production. As demonstrated in sensitivity analysis, fluctuations in NH_3_ spot price cause the most significant change in LCOH. With the assumption of an ideal treatment cost of $1.10 kg_H2_^−1^, achieving NH_3_ price below $310 ton^−1^ can bring LCOH down to $2.84 kg_H2_^−1^, making it comparable to the H_2_ from fossil‐fuel while with less CO_2_ emissions. Moreover, electrochemical NH_3_ synthesis has garnered increasing attention these years [99]. Despite limited to laboratory‐scale demonstrations, this technology enables the production of green NH_3_ (and consequently green H_2_) while drastically reducing the reliance on fossil fuels. By integrating NH_3_ e‐cracking, green hydrogen can even be produced and utilized in decentralized scenarios. By employing green NH_3_ as feedstock, the entire process of using NH_3_ as a H_2_ carrier can be realized with minimal CO_2_ emissions.
(vi) Strengthening international collaboration for scalable deployment of e‐cracking. Given the strong dependence of renewable energy availability on geography, establishing strategic export‐import partnerships for low‐carbon NH_3_ trade is crucial. Countries with locational advantages (e.g., Chile and Saudi Arabia) can form agreements with hydrogen‐consuming nations such as Japan and Germany to facilitate infrastructure development and investment. Furthermore, since commercially validated and standardized e‐cracking protocols are currently lacking, countries and research institutes should collaborate to establish a global NH_3_ e‐cracking standard, similar to the ISO 22734 framework for water electrolysis. This standard should define key factors such as efficiency benchmarks, NH_3_ leakage management, and acceptable byproduct limits. Such efforts will significantly accelerate the commercialization and large‐scale adoption of e‐cracking technologies.

By devoting efforts to these aspects for NH_3_ electrochemical cracking, NH_3_ can be more effectively utilized as a sustainable hydrogen carrier, marking a significant step toward the realization of a global green hydrogen economy.

## Author Contributions

**Yike Ye**: methodology, writing – original draft, data curation, validation, investigation, visualization, formal analysis. **Yi Shun Lim**: methodology, writing – original draft, data curation, investigation. **Qian Wu**: writing – review and editing. **Kamal Elouarzaki**: project administration, funding acquisition, writing – review and editing. **Zhichuan J. Xu**: conceptualization, project administration, supervision, resources, writing – review and editing, funding acquisition.

## Funding

This research is supported by the National Research Foundation, Singapore, and A*STAR (Agency for Science, Technology and Research) Under Its LCER Phase 2 Programme Hydrogen & Emerging Technologies FI, Directed Hydrogen Programme (Award No. U2305D4003).

## Conflicts of Interest

The authors declare no conflicts of interest.

## Supporting information




**Supporting File**: advs78256‐sup‐0001‐SuppMat.docx.

## Data Availability

The data that support the findings of this study are available from the corresponding author upon reasonable request.
