# Supplementary material for: Hydrogen Production from Ammonia Electrochemical Cracking
Source: Adv Sci (Weinh). 2026 Oct 11:e78256. Online ahead of print. doi: 10.1002/advs.78256 (PMC13655319; doi:10.1002/advs.78256)
Supplement: Supplementary file 1 [file ADVS-9999-e78256-s001.docx]

Supporting Information

**Hydrogen Production from Ammonia Electrochemical Cracking**

*Yike Ye, Yi Shun Lim, Qian Wu, Kamal Elouarzaki, and Zhichuan J. Xu**

1. **Energy Efficiency Calculations**

The energy efficiency *η* of NH_3_ E-cracking is determined as produced hydrogen energy divided by total energy input, which includes chemical energy of NH_3_ and electrical energy. Assuming 2 mol NH_3_ is E-cracked at a varying potential *U* from 0.1 V to 1.2 V, *η* is calculated as

$$\begin{aligned} \eta=\frac{\text{Hydrogen Energy}}{\text{Total Energy Input}}=\frac{3 mol\times241.9 kJ mol^{-1}}{U\times6 mol\times96485 C mol^{-1}+ 2 mol\times316.8 kJ mol^{-1}}\#\#\#\#(S1) \end{aligned}$$

Here, the lower heating values (LHVs) of H_2_ and NH_3_ are 241.9 kJ mol^-1^and 316.8 kJ mol^-1^, respectively. Faradaic constant is estimated to be 96485 C mol^-1^.

2. **Techno-economic Assessment (TEA) of E-cracking**

The H_2_ production scale is set to 1500 kg day^-1^, following hydrogen analysis (H2A) hydrogen production models.^[1]^ Spot price of NH_3_ fluctuates from $250 ton^-1^ to $750 ton^-1^, according to International Energy Agency (IEA).^[2]^ Therefore, NH_3_ spot price is set to $500 ton^-1^ in this study. In addition, the price of renewable electricity is assumed to be 0.03 $/kWh.^[2]^

*2.1. CAPEX*

2.1.1. Stack Cost Assessment

To illustrate the influence of catalyst price, we adopted assumptions from Jiao’s group^[3]^ and recalculated the stack cost: 1) The reference electrolyzer operates at 1.75 V and 0.4 A cm^-2^ with a stack cost of 342.11 $ kW^-1^ ($2016). 2) The active area of the reference electrolyzer is 3125 cm^2^. 3) Both cathode and anode originally took up 25% of total stack cost. 4) The mass loading of electrolyzers is 2 mg cm^-2^. Moreover, cathode catalyst is commercial 10 wt% Pt/C costing 3.2 $ g^-1^, and installation factor is 1.12. We also applied Chemical Engineering Plant Cost Index (CEPCI) to consider the cost change over the years, where CEPCI_2016_=541.7 and CEPCI_2025_=799.1. We note that CEPCI_2025_ is calculated by averaging the indices of first 6 months in 2025.

$$\begin{aligned} Electrode cost =\$\left( Cathode+Anode \right) g^{-1}\times Mass loading \times Active area \\ \#\left( S2 \right) \end{aligned}$$

$$\begin{aligned} Electrolyzer cost=342.11 \$ kW^{-1}\times0.5 + Electrode cost\#\left( S3 \right) \end{aligned}$$

$$\begin{aligned} Electrolyzer cost per area \left( \$ m^{-2} in 2025 \right)=Electrolyzer cost\times1.75 V \\ \times0.4 A cm^{-2}\times1.12\times\frac{799.1}{541.7}\times\frac{{10}^{4} cm^{2}}{m^{2}}\times\frac{kW}{1000W}\#\left( S4 \right) \end{aligned}$$

Using catalyst price in Table 1, we estimated electrolyzer cost per area of Pt_9_Ir, PtIr, and NiCu/NF is 2220.41, 3685.52 and 906.99 $ m^-2^ ($2025). Afterward, we can calculate stack cost by multiplying electrolyzer cost per area and overall required electrolyzer area estimated from the performance data. In detail, we assumed 100% Faradaic efficiency is achieved for HER. Then the daily current required can be calculated as

$$\begin{aligned} Current=\frac{1500 kg day^{-1}}{2.02\times{10}^{-3} kg mol^{-1}}\times2 mol\times96485 C mol^{-1}\times\frac{day}{86400 s}=1658502 A \# \end{aligned}$$

$$\left( S5 \right)$$

$$\begin{aligned} Stack cost=Electrolyzer cost per area\times\frac{Current}{Current density}\#\left( S6 \right) \end{aligned}$$

2.1.2. Balance of Plant

In addition to stack cost, CAPEX also includes expenditure of balance of plant (BoP), the auxiliary systems and components for power plant operations. Based on H2A model, we included mechanic BoP and electrical BoP with installation factors of 1.00 and 1.12, respectively. The corresponding expenses are 136.08 $ kW^-1^ and 121.00 $ kW^-1^ ($2016).^[1]^

$$\begin{aligned} Electricity=Current \times Voltage\#\left( S7 \right) \end{aligned}$$

$$\begin{aligned} Mechanic BoP=136.08 \$ kW^{-1}\times Electricity\times1.00\times\frac{799.1}{541.7}\#\left( S8 \right) \end{aligned}$$

$$\begin{aligned} Electrical BoP=121.00 \$ kW^{-1}\times Electricity\times1.12\times\frac{799.1}{541.7}\#\left( S9 \right) \end{aligned}$$

$$\begin{aligned} CAPEX= Stack cost+Mechanic BoP+Electrical BoP\#\left( S10 \right) \end{aligned}$$

2.1.3. Annuitized CAPEX

Assuming a factory lifetime *n* of 20 years and an interest rate *r* of 5%, we can calculate the equivalent annual cost (CAPEX_annual_) by applying annuity theory. Here, CRF stands for capital recovery factor (CRF).

$$\begin{aligned} CRF=\frac{r\times\left( 1+r \right)^{n}}{\left( 1+r \right)^{n}-1}=0.080\#(S11) \end{aligned}$$

$$\begin{aligned} {CAPEX}_{annual}=CRF\times CAPEX\#(S12) \end{aligned}$$

*2.2. OPEX*

2.2.1. Electricity

Electricity cost per day is calculated as follows.

$$\begin{aligned} Electricity cost=0.03 \$ k{Wh}^{-1}\times24 h\times Electricity\#\left( S13 \right) \end{aligned}$$

2.2.2. Feedstock

The feedstock cost is mainly constituted by NH_3_ consumption. Consequently, feedstock cost can be calculated as

$$NH_{3} consume rate=\frac{1500 kg}{2.02 g mol^{-1}}\times\frac{2}{3}\times17.03 g mol^{-1}=8430.7 kg day^{-1}$$

$$\begin{aligned} \#\left( S14 \right) \end{aligned}$$

$$\begin{aligned} Feedstock cost=8430.7 kg day^{-1}\times500 \$ ton^{-1}=4215.35 \$ day^{-1}\#\left( S15 \right) \end{aligned}$$

2.2.3. Maintenance

Assuming that the plant operates 350 days per year and that maintenance cost every year is 2.5% of overall CAPEX, then the maintenance cost per day is calculated as follows.

$$\begin{aligned} Maintenance cost=20 year\times{CAPEX}_{annual}\times2.5\%/(350 day)\#\left( S16 \right) \end{aligned}$$

2.2.4. Cell Compartment replacement

Cells must be replaced every 7 years to ensure a stable H_2_ production. The replacement fee is estimated as 15% of stack cost.^[3]^ Then the daily averaged cost of the replacement can be calculated as

$$\begin{aligned} Cell replacement=15\%\times s\mathrm{tack} cost / \left( 7\times365 day \right)\#\left( S17 \right) \end{aligned}$$

*2.3. Overall LCOH calculation*

LCOH can be calculated as follows:

$$LCOH=\frac{\frac{CAPEX_{annual}}{350 day}+Electricity+Feedstock+Maintenance+Cell replacement}{1500 kg day^{-1}}$$

$$\begin{aligned} \#\left( S18 \right) \end{aligned}$$

The results are presented in Table S1 below.

Table S1. LCOH and its components for catalysts. (Unit: $ kg_H2_^-1^)

| Catalyst | Pt_9_Ir | PtIr | NiCu/NF | Pt_9_Ir |
| --- | --- | --- | --- | --- |
| Feedstock | 2.81 | 2.81 | 2.81 | 2.81 |
| Annualized CPAEX | 0.81 | 1.07 | 0.29 | 0.25 |
| Electricity | 0.44 | 0.32 | 1.35 | 0.64 |
| Maintenance | 0.41 | 0.53 | 0.14 | 0.12 |
| Stack replacement | 0.20 | 0.27 | 0.05 | 0.05 |
| Poisoning loss | 0.04 | / | / | / |
| LCOH | 4.71 | 4.99 | 4.64 | 3.87 |

3. **Techno-economic Assessment (TEA) of thermal cracking**

We followed model from Peter’s book,^[4]^ and the cost of purchasing equipment was adopted from Lee et al.^[5]^ To compare LCOH results with E-cracking, we scaled up the production from 150 Nm^3^/h to 700 Nm^3^/h (1510 kg/day), and OPEX was recalculated correspondingly. In terms of CAPEX, we applied the six-tenth rule to estimate the cost of equipment when increasing the daily production.

$$\begin{aligned} \frac{Current cost}{Original cost}=\left[ \frac{Current scale}{Original scale} \right]^{0.6}\#\left( S19 \right) \end{aligned}$$

Table S2. CAPEX of base and burner model for thermal cracking.

|  | Percentage of fixed capital investment (FCI)/% | Base case/$ | With Burner/$ |
| --- | --- | --- | --- |
| **Direct Costs** |  |  |  |
| Purchased equipment | 42.8 | 622818.00 | 804218.00 |
| Equipment installation | 6.4 | 93131.66 | 120256.90 |
| Instrumentation  and control | 12.4 | 180442.60 | 232997.74 |
| Piping | 9.7 | 141152.68 | 182264.36 |
| Electrical | 5 | 72759.11 | 93950.70 |
| Building  (including services) | 3.6 | 52386.56 | 67644.50 |
| Total | 79.9 | 1162690.61 | 1501332.20 |
| **Indirect costs** |  |  |  |
| Engineering | 8.9 | 129511.22 | 167232.25 |
| Construction expenses | 9.7 | 141152.68 | 182264.36 |
| Legal expenses | 1.5 | 21827.73 | 28185.21 |
| Total | 20.1 | 292491.63 | 377681.82 |
| FCI for 150 Nm^3^/h | 100 | 1455182.24 | 1879014.02 |
| FCI for 700 Nm^3^/h |  | 3667089.04 | 4735153.79 |
| Working capital | 10 | 366708.90 | 473515.38 |
| Total CAPEX  for 700 Nm^3^/h |  | 4033797.94 | 5208669.17 |
| Annualized CAPEX |  | 323682.38 | 417957.09 |

Table S3. OPEX per day of base and burner model for thermal cracking.

|  | Base case/$ | With Burner/$ | Note |
| --- | --- | --- | --- |
| Feedstock | 5607.64 | 5607.64 |  |
| Cooling water | 80.87 | 80.87 |  |
| Electricity | 404.88 | 216.72 |  |
| Maintenance (M) | 209.55 | 270.58 | 2% of FCI |
| Operating supplies | 20.95 | 27.06 | 10% of M |
| Total OPEX | 6323.89 | 6202.86 |  |

**References**

[1]	G. S. M. Penev, C. Hunter, J. Zuboy, H2A Hydrogen Production Model: Version 3.2018 User Guide, <https://www.nrel.gov/hydrogen/assets/pdfs/h2a-production-model-version-3-2018-user-guide-draft.pdf>, accessed.

[2]	IEA, Ammonia Technology Roadmap. IEA: Paris, **2021**.

[3]	H. Shin, K. U. Hansen, and F. Jiao, "Techno-economic assessment of low-temperature carbon dioxide electrolysis," *Nature Sustainability* 4, no. 10 (2021): 911.

[4]	M. S. Peters, and K. D. Timmerhaus, *Plant design and economics for chemical engineers*, **1968**.

[5]	J. Lee, S. Ga, D. Lim, et al., "Carbon-free green hydrogen production process with induction heating-based ammonia decomposition reactor," *Chemical Engineering Journal* 457, (2023): 141203, <https://doi.org/https://doi.org/10.1016/j.cej.2022.141203>.
